# Improving continuity of patient care across sectors: study protocol of a quasi-experimental multi-centre study regarding an admission and discharge model in Germany (VESPEERA)

**DOI:** 10.1186/s12913-019-4022-4

**Published:** 2019-03-29

**Authors:** Johanna Forstner, Cornelia Straßner, Aline Kunz, Lorenz Uhlmann, Tobias Freund, Frank Peters-Klimm, Michel Wensing, Stephanie Kümmel, Nadja El-Kurd, Ronja Rück, Bärbel Handlos, Joachim Szecsenyi

**Affiliations:** 1 Department for General Practice and Health Services Research, University Hospital Heidelberg, Im Neuenheimer Feld 130.3, 69120 Heidelberg, Germany; 2 Department for Medical Biometry, University Hospital of Heidelberg, Institute for Medical Biometry and Informatics, Im Neuenheimer Feld 130.3, 69120 Heidelberg, Germany; 3 aQua -Institute GmbH, Maschmühlenweg 8-10, 37073 Göttingen, Germany; 4 AOK Baden-Württemberg, Presselstraße19, 70191 Stuttgart, Germany; 5 HÄVG Hausärztliche Vertragsgemeinschaft Aktiengesellschaft Regionaldirektion Süd, Kölner Str. 18, 70376 Stuttgart, Germany; 6 Gesundheitstreffpunkt Mannheim, Max-Joseph-Str. 1, 68167 Mannheim, Germany

**Keywords:** Patient admission, Patient discharge, Cross-sectoral care, Continuity of patient care, Patient readmission, Communication, Interprofessional coordination, Patient-centered care, Health services research, Implementation science

## Abstract

**Background:**

Hospitalisations are a critical event in the care process. Insufficient communication and uncoordinated follow-up care often impede the recovery process of the patient resulting in a high number of rehospitalisations and increased health care costs. The overall aim of this study is the development, implementation and evaluation of a structured programme (VESPEERA) to improve the admission and discharge process.

**Methods:**

We will conduct an open quasi-experimental multi-centre study with four intervention arms. A cohort selected from insurance claims data will serve as a control group reflecting usual care. The intervention will be implemented in 25 hospital departments and 115 general practices in 9 districts in Baden-Wurttemberg. Eligibility criteria for patients are: age > 18 years, hospital admission or hospitalisation, insurance at the sickness fund “AOK Baden-Wurttemberg”, enrolment in general practice-centred care contract. Each study arm will receive different intervention components based on the point of study enrolment and the patient’s medical need. The interventions comprise a) a structured assessment in the general practice prior to admission resulting in an admission letter b) a discharge conversation by phone between hospital and general practice, c) a structured assessment and care plan post-discharge and d) telephone monitoring for patients with a high risk of rehospitalisation. The assessments are supported by a software tool (“CareCockpit”), originally developed for structured case management programmes. The primary outcome (rehospitalisation due to the same indication within 90 days) and a range of secondary outcomes (rehospitalisation due to the same indication within 30 days; hospitalisations due to ambulatory care-sensitive conditions; delayed prescription of medication and medical products/ devices and referral to other health practitioner/s after discharge; utilisation of emergency or rescue services within 3 months; average care cost per year and patient participating in the VESPEERA programme) and quality indicators will be determined based on insurance claims data and CareCockpit data. Additionally, a patient survey on satisfaction with cross-sectoral care and health related quality of life will be conducted.

**Discussion:**

Based on the results, area-wide implementation in usual care is well sought. This study will contribute to an improvement of cross-sectoral care during the admission and discharge process.

**Trial registration:**

DRKS00014294 on DRKS / Universal Trial Number (UTN): U1111–1210-9657, Date of registration 12/06/2018.

## Background

Hospital discharges are a critical moment in healthcare delivery as continuity of care is crucial for patient safety. In daily practice, patients are often discharged at short notice, resulting in a lack of coordination of follow-up care and sufficient communication between care providers of the inpatient and outpatient sector and to the patient [1–3]. This can be due to different organisational procedures and different views on the patient and responsibilities with regard to the care process between hospitals and general practices [4] as well as between interprofessional teams [5]. The resulting interruptions in the continuity of care can affect the recovery process and healthcare related patient satisfaction. At worst, they can lead to avoidable rehospitalisations and adverse events [1, 4], which are relevant clinical outcomes and associated with increased care costs.

A project on harmonisation of admission and discharge medication showed the need for improvement of information brokerage concerning pre- and post- hospital medication as well as structured medication counseling after discharge [3].

Structured admission and discharge management are essential and inseparable components of optimal transitional care. Numerous systematic reviews provide overviews on evaluated transitional care programmes. For example, Leppin et al. found that a multicomponent intervention comprising many care providers with components such as case management, a telephone follow-up, patient-centred discharge information, and patient education significantly reduced readmission rates for medical and surgical patients [6]. In another review, Branowicki and colleagues implemented post-discharge interventions like follow-up phone calls, home visits, and discharge education [7]. The intervention showed that home visits and having two or more follow-up phone calls significantly reduced readmission rates. Vedel et al. conducted a review with studies focusing on transitional care interventions for patients with heart failure [8]. Intervention components included in the studies were telephone follow-up, individualised treatment plans, and medication schedules. The authors found that high intensity interventions significantly reduced readmission rates. Furthermore, they found a significant effect on reduction of emergency department visits. The results of Hansen et al. indicate that early discharge planning, follow-up phone calls and patient-centred discharge instructions may reduce 30-day rehospitalisation rates [9]. In another systematic review, Hesselink and colleagues gather studies that suggest structured discharge information, coordination of follow-up care and timely communication between care providers are components of successful discharge interventions [4]. Burke et al. found interventions in the domain “Systems for Monitoring and Managing Symptoms” of the Ideal Transitions Framework to be successful in reducing rehospitalisation rates [10]. Bahr et al. investigated post discharge telephone calls in single component interventions but could not find clear evidence on their effectiveness on reducing rehospitalisation rates and emergency department use and increasing patient satisfaction [11]. However, the authors suggest that further research should focus on telephone calls as a component of complex interventions involving outpatient care providers and high risk patients. In many systematic reviews, authors cannot draw firm conclusions, because the intervention components of the included studies are very heterogeneous and often lack comprehensible descriptions [4, 9, 12]. Nevertheless, some authors indicate that multicomponent interventions have a positive effect on outcomes such as rehospitalisation rates, emergency department visits, patient satisfaction, or health care cost [4, 9].

To date, in Germany there are few established standards for cross-sectoral communication related to arranged admission and discharge planning. Recent changes to German federal laws and regulations aim to improve transitions between care sectors. For example, the Act to Enhance Competition in Statutory Health Insurance (*Gesetz zur Stärkung des Wettbewerbs in der gesetzlichen Krankenversicherung*) aims to improve quality and efficiency of health care by enhancing competitions amongst care providers and sickness funds. A further example is the Act to Act to Promote Health Care in Statutory Health Insurance (*Gesetz zur Stärkung der Versorgung in der gesetzlichen Krankenversicherung*), which aims to improve continuity of patient’s care when discharged from hospital to other care providers. Still, fragmentation in care remains a complex challenge. With effect as of October 1st 2017, hospitals in Germany are obligated to offer a structured discharge planning to all patients according to the framework contract regarding hospital discharge management (“*Rahmenvertrag über ein Entlassmanagement beim Übergang in die Versorgung nach Krankenhausbehandlung nach § 39 Abs. 1a S.9 Sozialgesetzbuch V*”). However, this framework contract does not demand to conduct pre-hospital interventions to improve the admission process.

In the presented study, a structured admission and discharge management programme (VESPEERA) including follow-up care in general practices will be tested, thus providing a patient-centred and quality optimising enhancement of usual care.

The overall aim of this study is the development, implementation and evaluation of several transitional care components in general practitioners (GP) and hospitals.

### Objectives

Overall, the VESPEERA program is expected to reduce the number of avoidable rehospitalisations and emergency care contacts, to improve patient safety and patient involvement, to reduce overuse, underuse and misuse of health care, to improve the continuity of care and to improve interprofessional and cross-sectoral communication between patients, hospitals, GP-practices and the sickness fund “Allgemeine Ortskrankenkasse (AOK) Baden-Wurttemberg”.

## Methods/design

**Fig. 1:**
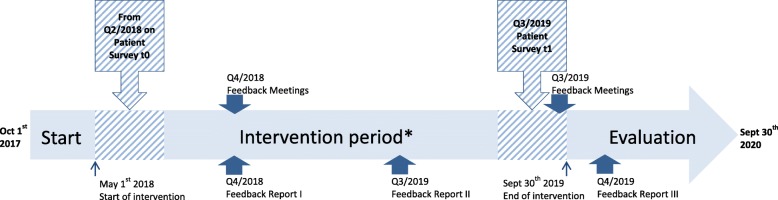
VESPEERA study timeline. Each study arm will receive different intervention components based on the point of study enrolment and the patient’s medical need, including structured assessment prior to admission and after discharge, automatically generated information for physicians and patients, a telephonic discharge conversation between hospital and general practice as well as a telephone monitoring for patients with a higher risk of rehospitalisation

An open prospective quasi-experimental trial will be conducted in multiple centres. Development of study material has started in October 2017. Recruitment of patients and implementation of study components (intervention phase) has started in May 2018 and will last through September 2019, comprising a total of 17 months. Participants can be enrolled until September 2019. All evaluations and publications are planned to be completed by the end of September 2020. Figure 1 illustrates the study timeline.

### Study setting

The VESPEERA programme will be implemented in 25 hospital departments and 115 general practices in 9 predefined geographical areas in the federal state Baden-Wurttemberg in South-Germany. Hospitals sign a participation contract according to §140a SGB V. The intervention region can be expanded if hospitals from other districts are willing to participate. However, hospitals outside the intervention region are not actively invited to participate. A sample of 50 GP-practices within the same federal state but outside the intervention region will serve as control sites for the patient survey.

### Study population / eligibility criteria

GP-practices within the intervention region have to participate in GP-centred care (*Hausarztzentrierte Versorgung/* HzV). GP-centred care is a programme where, amongst others, the GP has a gate-keeping function (further description of the programme provided by Wensing et al. [13]. Furthermore, Gp-practices have to employ a Care Assistant in General Practice (*Versorgungsassistentin in der Hausarztpraxis/* VERAH). Inclusion criteria for patients are age of 18 years and older, ability to give consent, insurance with the sickness fund AOK Baden-Wurttemberg, participation in GP-centred care according to § 73b SGB V, and at least one hospitalization during the intervention phase. Patients residing in long-term care facilities are excluded from study participation.

### Intervention group

The VESPEERA programme comprises recommended practices regarding discharge and care management, which is reinforced by laws (§ 11 Abs. 4 SGB V, § 39 Abs. 1a SGB V, § 112 Abs. 2, Satz 5 SGB V). The planned intervention picks up these regulations for optimal care and offers suggestions for their implementation and enhancement. Patients will not be randomly assigned to a study arm, but receive different intervention components based on the point of study enrolment and the patient’s medical need (see Table 1 – adjusted SPIRIT figure).

**Table 1:** Intervention components for all study arms

Interventions		*Study arm 1:*planned admission into a participating hospital	*Study arm 2:*planned admission into a non-participating hospital	*Study arm 3:*unplanned admission into a participating hospital	*Study arm 4:* unplanned admission into a non-participating hospital	*Study arm 5:* control group, not participating in VESPEERA
GP-practice	Interventions in the GP-practice before admission: (A) assessment for admission (B) admission letter and patient brochure	X	X			
Hospital	Interventions in the hospital: (C) telephonic discharge conversation (D) determination of HOSPITAL-Score and patient discharge information	X				
GP-practice	Interventions in the general practice after discharge: (E) assessment for planning of follow-up care (F) telephone monitoring, depending on the risk for rehospitalisation	X	X	X	X	

Assessment for Admission (A): Before a planned hospital admission, the VERAH collects patient information on basic data, admission diagnosis, medical history, existing prescriptions, medication plans and other medical information, social, living and legal situation, communication and care needs.

Admission letter and accompanying patient brochure (B): Based on the assessment for admission, an admission letter which also specifies how and when the practice is available by phone for the hospital staff is created. Patients receive a brochure that informs and prepares for the hospital stay.

Telephonic discharge conversation between GP-practices and hospital (C): For patients with complex interventions, medications or post-hospital care needs, a structured telephonic discharge conversation supports cross-sectoral exchange of information and clarification of responsibilities between the participating hospital and the responsible GP. In case of need, the AOK can be contacted to support the discharge process for example by providing medical products/ devices etc. in time.

Determination of HOSPITAL Score and patient discharge information (D): The participating hospitals collect data to determine the HOSPITAL Score, a score that indicates the risk for rehospitalisation within 30 days after discharge [14]. The score is communicated to the GP via the discharge letter. At the time of discharge, the patient receives patient discharge information, which summarizes the documents that the patient receives, follow-up care and appointments after discharge as well as different contact persons according to the respective care needs.

Assessment for planning of follow-up care (E): After hospital discharge, the patient has an appointment in the GP-practice (in case of need: home visit) with the VERAH. They conduct an assessment to determine the patients’ need for follow-up treatment and care. The GP receives a summary of the assessment, disclosing the patients’ care needs. Then, follow-up appointments, prescriptions and applications (such as rehabilitation or home care) can be determined and recorded. The patient receives a short summary of the arranged follow-up care plan. Based on the HOSPITAL Score, the GP determines the patients’ enrolment into the follow-up telephone monitoring. Patients with an intermediate or high risk for rehospitalisation, according to the HOSPITAL Score, should be enrolled in the follow-up telephone monitoring. For patients treated in a non-participating hospital, GP-practices have the option to gather data to determine the HOSPITAL Score. All data necessary to determine the score can be extracted from the discharge letter.

Post discharge telephone monitoring (F): If the HOSPITAL Score indicates an intermediate or high risk for rehospitalisation, the GP-practice coordinates and accompanies follow-up care through a structured telephone monitoring, which is limited to a time frame of 3 months after hospital discharge. The GP individually defines the content and frequency of the telephone monitoring, which is executed by the VERAH. The first phone call should be scheduled within the first 2 weeks after discharge. The last phone call should be scheduled 3 months after discharge. Any phone calls in between are defined in their interval by the GP.

### Control group

Using propensity score matching a control group from non-participating HzV-GP-practices in non-participating districts is built. Using claims-based data, age, gender, practice size and length of enrolment in GP-centred care and the district are considered to build the control group on practice level; to build a patient control group, age, gender, indication group and Charlson Comorbidity Index are used. Patients in the control group are not participating in the study and do not receive any intervention components.

The control group for the patient survey comprises patients that from approx. 50 comparable HzV-practices outside the intervention region. The inclusion criteria of the intervention group apply for patients of the control group.

### Implementation strategies

Several strategies have been chosen in order to support the implementation of the VESPEERA programme. First, the CareCockpit software is used, providing a platform to GP-practices that assists with organising patient information, organising care planning, guidance through assessments and automatic generation of documents. The CareCockpit is routinely used in Baden-Wurttemberg within the practice-based case management programme PraCMan (*Hausarztpraxis-basiertes Case Management*)) and is now enhanced by an additional module for the VESPEERA programme [15]. GP-practices receive the CareCockpit-software free of charge. Second, representatives of all stakeholders, thus clinicians, GPs, patients, sickness funds and researchers, have been involved in the development of assessment instruments and study documents in order to increase acceptance and utilisation. In several workshops the intervention components and all their items were discussed with regard to its relevance, information value, feasibility, and degree of sensitivity as well as wording. Third, GPs and VERAHs will participate in a workshop to be instructed in software utilisation and study processes. The workshops are delivered in a train-the-trainer format, i.e. the study team trains a certain number of GPs who then lead regional workshops for their participating colleagues. Fourth, participating hospitals will hand in a description of their individual implementation plan. In order to facilitate the integration of study components into clinical processes, different approaches are suitable for different hospitals. Therefore, each hospital will provide information on how they will ensure the identification of study patients, the use of the admission letter, the execution of the telephonic discharge conversation, the dissemination of the patient discharge information, and the determination of the HOSPITAL Score. Hospitals can choose to either integrate the intervention components into their medical information system or to choose a paper-based solution, depending on their processes. Additionally, hospitals are offered two options to provide the patient discharge information: (a) hospitals use the template provided by the study team or (b) hospitals integrate the elements of the study template into their own documents. Fifth, all stakeholders will receive feedback reports in form of benchmarking reports in September 2018, June 2019 and December 2019. The feedback reports are based on claims data, data from the CareCockpit and patient survey data, aggregated on a hospital / GP-practice level. The feedback reports will be discussed in moderated interdisciplinary feedback meetings. GPs, representatives of the hospitals as well as patients will participate in these meetings and discuss options for potential improvement. Additionally, hospitals and GP-practices will receive expense allowances for conducting patient-related care services as well as lump sums for study organisation and participation in workshops and feedback meetings.

### Data sources

Data sources for the evaluation are claims-based data from AOK Baden-Wurttemberg, data from the CareCockpit software, primary data in the form of patient surveys in the intervention group as well as a control group, and data collected in participating hospitals in order to determine the HOSPITAL Score. This includes haemoglobin and sodium levels, length of stay, type of admission (elective vs. non-elective), discharge from an oncology service or execution of an oncological treatment, any OPS-coded procedure and number of hospital stays in the last 12 months.

CareCockpit data includes the pseudonym generated for data linkage, diagnoses, the medical question for admission, information on previous antibiotic prescriptions, living situation, long-term care related items (such as ADL and IADL-scale), medical information (such as pain, wounds, alarming symptoms for medical emergencies, PHQ-2 instrument), compliance to medicinal therapy, the items of the HOSPITAL Score as well as process data (provision of information to patients, information on whether any follow-up care has been initiated and successfully executed).

The patient survey questionnaire includes the following instruments: sociodemographic questions, the validated EQ-5D for health related quality of life [8] as well as selected items from the validated PEACS-instrument for satisfaction with cross-sectoral care concerning the admission and discharge process [16]. Patient surveys are conducted in GP-practices belonging to the intervention group as well as GP-practices that build a control group with all patients who, in the first (t0) and in the last 3 months of the intervention phase (t1) respectively come into their GP-practice after a hospital stay. The intervention phases can vary between the GP-practices of the intervention group depending on when the respective practice actually started its participation in VESPEERA.

### Outcomes

The primary outcome is the number of rehospitalisations due to the same indication (three-digit ICD-10-GM code) within a time frame of 3 months (90 days) to the outpatient sector. This outcome is collected from claims-data on a case level, meaning that each patient can have more than one rehospitalisation, whereof all are considered in the analyses.

The following indicators have been defined as secondary outcomes and are based on pseudonymised claims-data, data collected within the CareCockpit software in GP-practices as well as patient survey data:

1. number of rehospitalisations due to the same indication (three-digit ICD-10-GM code) within a time frame of 30 days
2. number of hospitalisations due to ambulatory care-sensitive conditions [17]
3. number of patients discharged to the outpatient sector from a participating hospital where the prescription of medication is delayed [18]
4. number of patients discharged to the outpatient sector from a participating hospital where referral to other health practitioner/s (*Heilmittel*) is delayed
5. number of patients discharged to the outpatient sector from a participating hospital where prescription for medical products/devices (*Hilfsmittel*) is delayed
6. number of patients discharged from a participating hospital who use emergency or rescue services (emergency medical service (*Ärztlicher Bereitschaftsdienst*), emergency ambulance (Notarztwagen), ambulance emergency response vehicle (*Notarzteinsatzfahrzeug*), ambulance, rescue helicopter (*Primärtransport – Luft*) within a time frame of 3 months
7. average care cost per year and patient participating in the VESPEERA programme
8. patient-reported experiences and health-related outcome of cross-sectoral care using the German version of the instrument Patients’ Experiences Across Health Care Sectors (PEACS) [16]
9. health-related quality of life, using the instrument EuroQol (EQ-5D) [19].

### Recruitment

All eligible GP-practices located within the predefined districts will be contacted by the HÄVG (*Hausärztliche Vertragsgemeinschaft,* HÄVG, the organisation that manages the GP-centred care contracts for the German Association of General Practitioners) via fax or on other communication channels such as newsletters and in continuing education workshops. If interested in participation, GPs and VERAHs sign up for the training described above. During the training they will receive detailed information about the study and sign the informed consent form.

Participating practices as study sites will check the inclusion criteria for a) patients with a planned hospital admission and b) patients after discharge after an unplanned hospital stay, provide the study information, and receive the signed informed consent forms which remain at the study site.

For the recruitment of the control group for the patient survey, GP-practices outside the intervention region will check the inclusion criteria for patients after hospital discharge. They hand out the patient survey questionnaire to eligible patients. Patients give their consent by filling in the anonymous questionnaire. Controls for the patient survey are recruited by GP-practices outside the intervention region.

### Sample size calculation

**Fig. 2:**
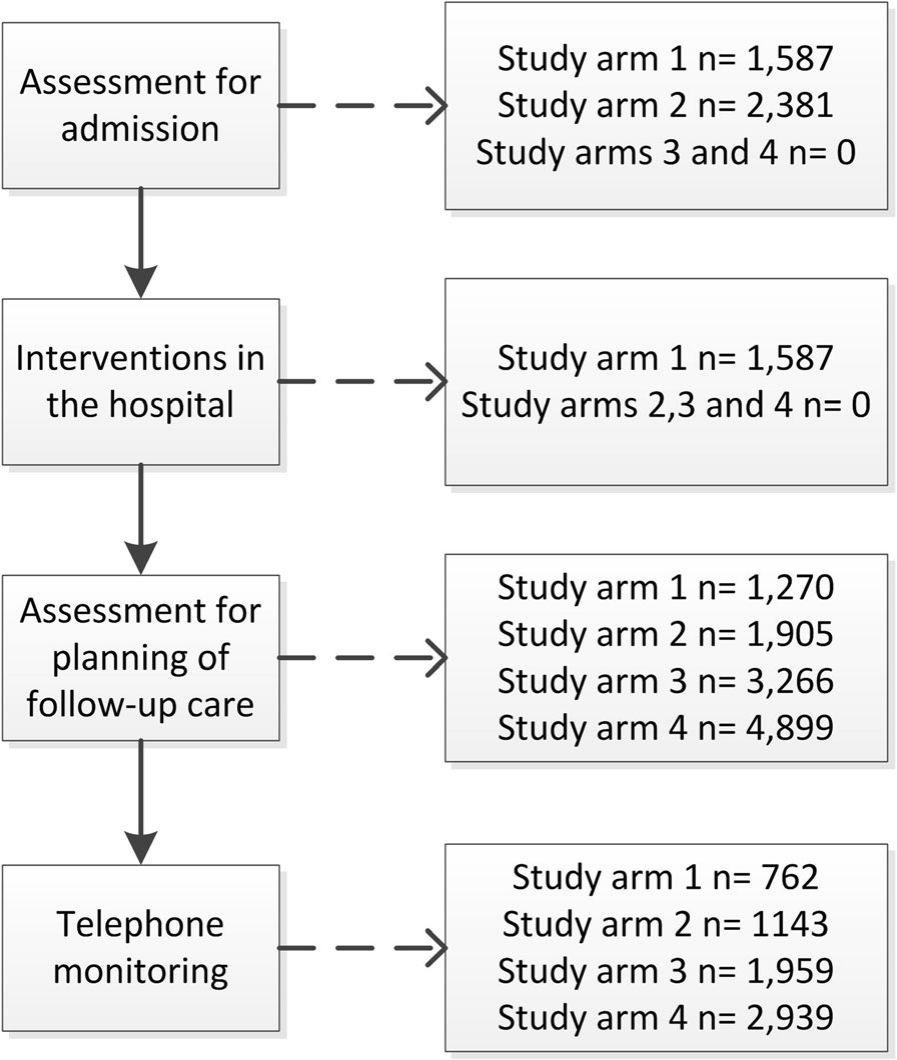
Expected sample sizes in all four study arms

Since large-scale implementation of the VESPEERA program in a geographical region was intended, the number of participating GPs and hospitals was not restricted. Based on the number of GP-practices who participate in HzV-Care and the number of GP-practices who participate in the PraCMan programme, a number of 115 GP-practices are expected to participate. An estimation of the patient sample size was made using claims-data and several assumptions for hospitalisation rates, rates for planned and unplanned admissions, rates of patients with a high risk of rehospitalisation as well as drop-out rates. Assumptions were made based on analyses of AOK claims-data. Admissions are expected to be planned and unplanned in 50% of the cases, respectively. 56% of planned admissions are made by a GP and therefore can be included in the VESPEERA programme. 40% of planned admissions are cases that are expected to be treated in a participating hospital department. After discharge, we calculated with a 20% drop-out rate for patients who are transferred into a nursing home, who pass away or who do not seek GP contact after discharge. 60% of discharged patients are expected to have a risk of rehospitalisation (HOSPITAL Score indicating an intermediate or high risk for rehospitalisation). Figure 2 gives an overview on the sample sizes in each study arm. Given the estimated sample size, we can estimate the effect size (based on the hospitalisation rates after the intervention) that can be detected. Based on claims-data different numbers of hospitalisations can be expected for each of the intervention groups. The alpha level is set to 5%. The following power considerations are based on chi square tests (to test for group differences) and adjusted for the hierarchical structure of the data. The implemented models in the primary analysis will most likely lead to even higher power values. However, we consider the hierarchical structure of the data with cases nested in patients nested in hospitals or practices, respectively. A relatively high ICC (0.3) can be expected when considering measurements within patients, however the number of cases per patient will be relatively small (approx. 1.6 cases per patient). The clustering by hospitals or practices is taken into account with an expected number of approx. 540 patients per hospital and a presumed ICC of 0.03, which is a realistic value in health services research [20]. Furthermore, we assume a rate of re-hospitalisations of 23% in the control group. With a power of 80%, the following reductions in each of the intervention groups can be shown: For study arm 1 a sample size of 1905 cases can be expected. Hereby, a reduction of approx. 8% (from 23% to approx. 15%) can be shown. The control group will be matched and, therefore, the sample size will be the same. For study arm 2 when compared to the control group, the same reduction is detectable, as the same number of cases can be expected as in study arm 1. In a last step, both the study arms 3 and 4 are compared to the control group using a level of significance of 2.5%. In study arm 3, we expect a sample size of 3266 and, thus, a reduction of approx. 7% (from 23 to 16%) can be shown. In study arm 4, a sample size of 4899 hospitalizations can be expected which allows to show a reduction of approx. 6% (from 23 to 17%).

### Data collection and management

Primary and secondary outcomes, as well as patient characteristics, are extracted from claims-data at the health insurer involved, CareCockpit data and primary data from a patient survey.

Claims-based data are collected for a time frame starting 6 months before baseline until 3 months after the intervention phase. Data from the CareCockpit is transferred along with administrative data each quarter year.

For the conduction of the patient survey, GP-practices are informed in advance on when to conduct the survey and they receive all material needed including the patient questionnaires, the sealed box to collect them, and detailed information on how to conduct the survey in the practice. The patients are asked in the practice by their physician after discharge to take part in the survey by filling in the questionnaire in the practice. Questionnaires will be handed out to all eligible patients per practice and time point in both intervention and control practices. The sealed boxes with filled-in questionnaires will be sent by mail using an enclosed post-paid box directly to the aQua-Institute.

The hospitals have the choice to either integrate the questionnaire into their hospital information system as an electronic questionnaire (transfer to the aQua-Institute via secure file transfer protocol (SFTP)-servers) or to fill in paper-based questionnaires that are sent to the aQua-Institute via mail using enclosed post-paid envelopes. If GP-practices are responsible for determining the HOSPITAL Score, the items of the HOSPITAL Score are part of the assessment for planning of follow-up care within the CareCockpit and will be transferred to the evaluating institutions along with other CareCockpit data. All data necessary to determine the score can be extracted from the discharge letter.

As the intervention is implemented in a pragmatic study design, differences in quality between the GP-practices and hospitals can occur. For quality assurance purposes, all GP-practices are therefore continuously being contacted via telephone by the study central office. Within the phone calls, recruitment status in the GP-practice is being checked and support with the implementation is offered. Additionally, any issues that occur with the implementation will be addressed in feedback meetings. Furthermore, the CareCockpit software guides through the assessments.

### Data analysis

The outcome evaluation is divided into a primary analysis and secondary analyses. Design and analysis of the claims-data based evaluation will closely follow a recently published US-study on reduction of rehospitalisations in high-risk patients [2]. They are well-grounded and established in the literature on programme evaluation, especially in econometrics and quantitative social science, and increasingly in health systems research [21–24].

#### Primary analysis

In the primary analysis, a difference-in-difference model is applied [21, 25, 26]. The change of the primary outcome (before vs. after the intervention) of each intervention group will be pairwise compared to the control group. Put in a simple way, the following model will be estimated:

$$ {\mathrm{rehospitalisation}}_{\mathrm{t},\mathrm{i}}={\upbeta}_0+{\upbeta}_{\mathrm{T}}\mathrm{x}\ {\mathrm{t}\mathrm{ime}}_{\mathrm{t},\mathrm{i}}+\kern0.37em {\upbeta}_{\mathrm{I}}\mathrm{x}\ {\mathrm{i}\mathrm{ntervention}}_{\mathrm{i}}+\kern0.37em {\upbeta}_{\mathrm{I}\mathrm{x}\mathrm{T}}\mathrm{x}\ {\left(\mathrm{intervention}:\mathrm{time}\right)}_{\mathrm{t},\mathrm{i}}. $$

The index t describes the point in time, i is the index for the considered observation. The following parameters will be estimated:

- β_0_: intercept
- β_T_: effect of time; the general change from before to after the intervention
- β_I_: difference between groups at baseline
- β_IxT_: difference between the change (before and after comparison) in the control and in the intervention group; the main parameter for the outcome evaluation

The assumptions of this model are sufficiently discussed in the literature. The parameter β_IxT_ is the main parameter to evaluate the effectiveness of the intervention, describing the difference in changes between the two groups over time.

The above model is adjusted for the covariate service level of the hospital and the location (general allocation to districts). Other covariates will be already considered during the matching procedure when generating the control group.

The primary outcome is binary. Thus, logistic regression models will be applied. Furthermore, there is a hierarchical structure underlying the data, with cases nested in patients, and patients nested in practices or hospitals, respectively. This structure is considered in the model by including random intercepts at each level. The combination of random and fixed effects results in a mixed logistic regression model. A compound symmetry correlation structure will be assumed when fitting the models.

In the primary analysis, one model per intervention arm will be applied which leads to the need of an adjustment of our testing procedure for multiple testing. In order to meet the global level of significance of 5%, the following procedure will be applied: Study arm 1 (planned admission into a participating hospital) is expected to show the strongest effect. Therefore, this comparison will be considered using the full α-level of 5%. If the null hypothesis of no intervention effect can be rejected, the second study arm is tested at the same level. In a last step, the study arms 3 and 4 are tested simultaneously by splitting the level of significance equally according to Bonferroni to 2.5% each.

#### Secondary analysis and sensitivity analysis

In addition to the primary analysis, further analyses will be conducted. First, again using the primary outcome but including 12 time points (six before the intervention and six after the intervention (measured month-wise), time trends before and after the intervention will be compared using interrupted time-series-models [27, 28]. Secondary endpoints will be analyzed using the same strategy as used in the primary analysis with adjustments of the models as necessary.

Furthermore, descriptive statistics of all collected data will be performed. Continuous variables will be described using means, standard deviations, median, minimum, maximum, and first and third quartile, separately for each intervention group and the control groups as well as for the total population. Categorical variables will be reported using absolute and relative frequencies.

As claims-based data are used, no missing values are expected to occur, therefore no imputation procedures need to be carried out.

#### Analysis sets

The primary analysis will be based on the intention-to-treat sample. This means that, in the analysis, patients will be considered in the treatment arm they were assigned to even if, for instance, not all interventions that were planned in this arm were delivered. In sensitivity analyses, the per-protocol set will be analyzed where these patients will be omitted. Patient to be excluded in this set will be defined before the analysis is performed. In a further as-treated-set the omitted patients will be included again but assigned to the treatment arm that describes best the treatment they actually received.

### Ethics, data protection and security and dissemination

The study protocol has been submitted to and approved by the ethics committee of the Medical Faculty Heidelberg as well as the ethics committee of the State Medical Council of Baden-Wurttemberg prior to the start of the study. Any amendments to the protocol are disseminated to GP-practices via post and have to be confirmed via fax, GP-practices will be called until the study central office receives a signature as confirmation.

The regulations of the Data Security Law of Baden-Wurttemberg (*Landesdatenschutzgesetz*), the German Federal Data Security Law (*Bundesdatenschutzgesetz*), respectively, are met. All responsibilities concerning data management and analysis as well as access to data is declared in a separate and elaborate data protection concept which is part of the contractual agreement between consortium partners and has been approved by a data security officer.

To enhance transparency and integrity, the SPIRIT checklist was used during compilation of this study protocol [29].

## Discussion

We expect to receive results on the effectiveness of the components included on the outcomes that guide the evaluation. Based on the results, we will adjust the intervention with regards to content and possibly define the scope of the patient collective that benefits most from the intervention. An implementation in area-wide usual care and hence standards for transitional care that involve care providers from both the inpatient and the outpatient sector is well sought. Thereby, we will be able to contribute to the scientific knowledge on admission and discharge management in general and especially in Germany.

We also hope to lay the foundation for bringing together stakeholders involved in cross-sectoral care in order to stimulate exchange and to offer a platform to collectively tackle the challenges of transitions in care.

However, this study has a number of limitations. The implementation of intervention measures means additional time expenditure for hospitals, GP-practices and patients. Therefore, the acceptance and intended provision of study components might be a critical factor. Nevertheless, we expect appointments especially within the GP-practice to be more structured and targeted. Acceptance of the intervention amongst others will be investigated in an accompanying process evaluation. A separate study protocol for the process evaluation will be handed in for publication.

Study results will be published in final reports to the funding agency, articles in peer-reviewed journals, and will be presented on national and, if eligible, international conferences. Furthermore, all participating GP-practices and hospital centers will receive feedback reports and the final report. A project-related website addresses any study-related content and serves as an instrument to communicate any news relating to the study.

### Trial status

The study protocol on hand is the protocol version 2.0 from July 30th 2018. Recruitment started on June 15th 2018 and will approx. be completed by the end of September 2019.

## Abbreviations

AOK: Allgemeine Ortskrankenkasse, large German sickness fund
GP: General practitioner
HÄVG: Hausärztliche Vertragsgemeinschaft
HzV: General practitioner centred-care programme(*Hausarztzentrierte Versorgung*)
PraCMan: General practice-based case management programme (*Hausarztpraxisbasiertes Case Management*)
SFTP: Secure File Transfer Protocol
VERAH: Care Assistant in General Practice (*Versorgungsassistentin in der Hausarztpraxis*)
